# Early life adversity increases foraging and information gathering in European starlings, *Sturnus vulgaris*

**DOI:** 10.1016/j.anbehav.2015.08.009

**Published:** 2015-11

**Authors:** Clare Andrews, Jérémie Viviani, Emily Egan, Thomas Bedford, Ben Brilot, Daniel Nettle, Melissa Bateson

**Affiliations:** a Centre for Behaviour and Evolution, Institute of Neuroscience and Newcastle University Institute of Ageing, Newcastle University, Newcastle upon Tyne, U.K.; b Département de Biologie, École Normale Supérieure de Lyon, Université de Lyon, Lyon, France; c School of Biological Sciences, Plymouth University, Plymouth, U.K.

**Keywords:** body mass regulation, contrafreeloading, developmental stress, early life adversity, European starling, food insecurity, foraging, *Sturnus vulgaris*

## Abstract

Animals can insure themselves against the risk of starvation associated with unpredictable food availability by storing energy reserves or gathering information about alternative food sources. The former strategy carries costs in terms of mass-dependent predation risk, while the latter trades off against foraging for food; both trade-offs may be influenced by an individual's developmental history. Here, we consider a possible role of early developmental experience in inducing different mass regulation and foraging strategies in European starlings. We measured the body mass, body condition, foraging effort, food consumption and contrafreeloading (foraging for food hidden in sand when equivalent food is freely available) of adult birds (≥10 months old) that had previously undergone a subtle early life manipulation of food competition (cross-fostering into the highest or lowest ranks in the brood size hierarchy when 2–12 days of age). We found that developmentally disadvantaged birds were fatter in adulthood and differed in foraging behaviour compared with their advantaged siblings. Disadvantaged birds were hyperphagic compared with advantaged birds, but only following a period of food deprivation, and also spent more time contrafreeloading. Advantaged birds experienced a trade-off between foraging success and time spent contrafreeloading, whereas disadvantaged birds faced no such trade-off, owing to their greater foraging efficiency. Thus, developmentally disadvantaged birds appeared to retain a phenotypic memory of increased nestling food competition, employing both energy storage and information-gathering insurance strategies to a greater extent than their advantaged siblings. Our results suggest that subtle early life disadvantage in the form of psychosocial stress and/or food insecurity can leave a lasting legacy on foraging behaviour and mass regulation even in the absence of food insufficiency during development or adulthood.

## Highlights

• Starvation may be avoided by storing energy reserves or gathering information.
• Developmental history could impact these foraging decisions.
• Starlings disadvantaged in nestling competition were fatter in adulthood.
• Developmentally disadvantaged birds foraged faster and contrafreeloaded more.
• Early life stress has a lasting legacy on foraging behaviour and mass regulation.

Food availability is often unpredictable in the wild. One means for animals to reduce the risk of starvation is to store energy reserves, while another is to gather information about alternative food sources which could be useful later (Dall & Johnstone, 2002; Mathot & Dall, 2013). Animals facing increased starvation risk or reduced access to food due to competition maintain more stored reserves (Houston & McNamara, 1993; Witter & Swaddle, 1995), which they must forage to obtain. However, they must trade off carrying reserves against increased costs of locomotion or mass-dependent predation (Macleod, Gosler, & Cresswell, 2005). Environmental stochasticity also impacts optimal sampling of the environment to gain information (Dunlap & Stephens, 2012; Keasar, Motro, & Shmida, 2013; Shettleworth, Krebs, Stephens, & Gibbon, 1988). With limited time and energy to invest, animals will also face a trade-off between the need to gain food and the need to gather information about alternative food sources. These trade-offs governing foraging for food or gathering information, and storing energy reserves, may be influenced by developmental history. For example, developmental history may impact locomotor ability (O'Hagan, Andrews, Bedford, Bateson, & Nettle, 2015) and hence predation risk. Understanding the impact of early environmental conditions on foraging decisions and information use is critical to evolutionary biologists studying phenotypic plasticity and the causes of individual behavioural differences (Dall, Bell, Bolnick, & Ratnieks, 2012; Mathot & Dall, 2013). It may also aid understanding of the developmental origins of food-related conditions such as obesity and metabolic syndrome in humans (e.g. Cottrell & Ozanne, 2008; Gluckman & Hanson, 2007; Khlat, Jusot, & Ville, 2009; Levin, 2006). We consider here the possible role of early developmental history in altering an individual's mass regulation, foraging behaviour and information gathering.

Optimal foraging theory (Stephens & Krebs, 1986) predicts that animals should preferentially forage on patches offering the highest net rate of energy intake. Paradoxically, in many species including humans, animals sometimes choose to forage in patches in which effort is required to exploit the food resource despite the existence of alternative patches containing identical food available for minimum effort (Coulton, Waran, & Young, 1997; Inglis, Forkman, & Lazarus, 1997; Larson & Tarte, 2013; Lindqvist & Jensen, 2008; Neuringer, 1969; Ogura, 2011; Robertson & Anderson, 1975; Vasconcellos, Harumi, & Ades, 2012). This behaviour whereby animals work for food even when the same food is freely available is known as contrafreeloading (CFL). The ‘information hypothesis’ offers a functional explanation for CFL, suggesting that it is a form of sampling to gather information that may be useful in future foraging attempts (Bean, Mason, & Bateson, 1999; Forkman, 1996; Inglis & Ferguson, 1985). Animals performing CFL demonstrate that they know the location of the most profitable foraging patches when the previously most profitable patch is removed, or while they are more hungry (Bean et al., 1999; Forkman, 1996; Lindqvist, Schütz, & Jensen, 2002). Current energetic state appears to affect the trade-off between foraging and gathering information, with food deprivation decreasing CFL in starlings (Bean et al., 1999; Inglis & Ferguson, 1985) and chickens, *Gallus gallus domesticus* (Lindqvist et al., 2002). Yet despite growing interest in the drivers of individual differences in information use (Mathot & Dall, 2013), the potential impact of past environment on this trade-off, and hence information gathering via CFL, has not yet been investigated.

Adverse environmental conditions experienced early in life can strongly impact adult survival and health, in humans and other animals (Lindström, 1999; Lummaa & Clutton-Brock, 2002). The relationship between prenatal or early postnatal environment and obesity is an active research field (Cottrell & Ozanne, 2008; Gluckman & Hanson, 2007; Khlat et al., 2009; Levin, 2006), although there has been limited attention on the evolutionary and associated proximate behavioural mechanisms involved. Evidence from rodent and avian models suggests that adult foraging decisions may be influenced by developmental history. Early food restriction of rodents induces an increased drive to obtain and consume food in adulthood, with rats, *Rattus norvegicus*, whose mothers were fed restricted diets in the perinatal period being hyperphagic long beyond weaning (Coupé, Grit, Darmaun, & Parnet, 2009; Orozco-Sólis et al., 2009; Qasem et al., 2012; Vickers et al., 2000). In starlings, increased competition for food in the nest affects foraging decisions in adulthood in terms of the trade-off between the benefits of obtaining nutrients and the costs of ingesting toxins by consuming toxic prey (Bloxham, Bateson, Bedford, & Nettle, 2014). The propensity to contrafreeload may also be impacted by growth early in life. In broiler chickens (selected for high feed conversion efficiency) a reduction in CFL compared with layer chickens or the ancestral red junglefowl, *Gallus gallus*, has been interpreted as a selected energy-saving response to the demands of rapid growth (Lindqvist & Jensen, 2008; Lindqvist et al., 2002; Lindqvist, Zimmerman, & Jensen, 2006; Schütz & Jensen, 2001). However, no study has yet experimentally investigated how developmental history may influence the amount of CFL individuals choose to perform.

In the current study, we used a recently developed subtle early life manipulation of food competition in wild European starlings to investigate the effect of early life adversity on adult foraging behaviour and body mass. The European starling is a species known to reliably display CFL, showing considerable individual variation in this behaviour (Bean et al., 1999; Inglis & Ferguson, 1985). To control for genetic effects, we cross-fostered siblings on day 2 of life until day 12, to a nest in which they were either slightly larger than the other chicks (the advantaged treatment) or slightly smaller (the disadvantaged treatment). Previous similar manipulations suggest that disadvantaged nestlings would have had to beg more in order to be fed, and would have been jostled to more peripheral positions in the nest (Cotton, Wright, & Kacelnik, 1999; Kacelnik, Cotton, Stirling, & Wright, 1995). Although our manipulation did not affect growth, there was evidence that it did increase developmental stress. Indeed, telomere attrition, an acknowledged biomarker of developmental stress exposure, was greater in the disadvantaged than the advantaged nestlings, yet they did not differ in growth (Nettle, Monaghan, et al., 2015). Following the manipulation, we brought the birds into captivity and reared them to adulthood under uniform conditions. When they were between 10 and 13 months of age we studied their energy reserves and foraging on freely available food versus contrafreeloading via searching for food hidden in sand.

In light of theory and previous findings in rodents, we predicted that developmentally disadvantaged birds would be hyperphagic and carry greater energetic reserves than the advantaged birds. Since our manipulation did not detectably impact growth (Nettle, Monaghan, et al., 2015), we might assume that developmentally disadvantaged starlings would behave unlike fast-growing broiler chickens (which show reduced CFL to conserve energy). Instead, we predicted that disadvantaged birds would show increased CFL as a means to gather information as insurance against future food insecurity.

## Methods

### Overview

Subjects were 37 European starlings (23 male, 14 female; 19 advantaged, 18 disadvantaged) from 10 natal families. These birds were subject to a developmental manipulation of early life competition as nestlings in the field (day 2–12 posthatching) whereby they were cross-fostered in sibling pairs into 20 different host nests in which they were either the smallest (disadvantaged treatment) or largest (advantaged treatment) nestlings in the brood size hierarchy, after which time they were raised under identical laboratory conditions. Birds were 10–13 months old at the time of the current experiment. For the experiment, birds were housed individually and pretrained to forage for food hidden in sand. Once a stable level of food consumption was achieved, birds entered an experimental phase during which we presented them with a choice between foraging for freely available food or identical food hidden in sand during a 2 h trial, daily for 4 days. We measured foraging effort and consumption during trials, as well as food consumption outside trials when food was freely available.

### Developmental Manipulation

The developmental manipulation is described in detail elsewhere (Nettle, Monaghan, et al., 2015). Briefly, in 2013 on posthatching day 2 (henceforth day 2), we removed 12 quartets of siblings from the natal nest and cross-fostered them to two different host nests per quartet; the two randomly selected nestlings in the advantaged (ADV) condition were fostered to a nest where they were (mean ± SD) 4.9 ± 1.9 g larger than all other nestlings, and the two in the disadvantaged (DIS) condition to a nest where they were 4.8 ± 2.2 g smaller than the other nestlings. The composed brood size ranged from four to six chicks but was matched for the ADV and the DIS half of a focal quartet. These size differences against an average day 2 weight of 13 g were less extreme than the average difference between the smallest and largest chicks in unmanipulated broods (10.4 ± 0.6 g against an average day 4 weight of 20 g; Andrews et al., n.d.). On day 12, cross-fostered nestlings were removed to the laboratory (complete surviving quartets only) where the natal families were reconstituted and nestlings hand-reared to independence at around 6 weeks of age (for details of hand-rearing methods see Feenders & Bateson, 2011), whereafter they lived in communal aviaries. ADV and DIS nestlings did not differ significantly in body mass at any age during development (measured at days 3, 4, 7, 12, 15, 18, 21 and 24), although the ADV nestlings remained significantly heavier than their unrelated host nest competitors at day 12, while the DIS nestlings remained significantly smaller than their host nest competitors (Nettle, Monaghan, et al., 2015). Wing lengths did not differ significantly by treatment at day 12 or after fledging on day 24 (Nettle, Monaghan, et al., 2015).

### Housing and Husbandry

When not in experimental procedures, we housed birds in mixed-sex groups of up to 20 in two indoor aviaries (215 × 340 cm and 220 cm high; ca. 18° C; 40% humidity; 15:9 h light:dark cycle). Birds were provided with environmental enrichment (foraging substrate, water baths, multilevel rope perches, suspended cardboard boxes as cover), clean drinking water, and were fed ad libitum on domestic chick crumb (Special Diets Services ‘Poultry Starter (HPS)’), supplemented with cat biscuits (Royal Canin Ltd. ‘Fit’), dried insect food (Orlux insect pâté), live mealworms and fruit. The birds were maintained in nonbreeding condition by means of an unchanging light:dark cycle of long days. For the experiment, birds were taken into the experimental laboratory in groups of eight, with all four members of a natal family in the same group. Birds were individually housed in wire-mesh cages (75 × 45 cm and 45 cm high) fitted with two wooden perches, two water bottles, and a central wooden divider (8.5 cm high) across the centre of the cage. Bowls of water for bathing were provided daily for 20 min. The birds were maintained under a 15:9 h light:dark cycle, at ca. 18° C; 40% humidity. Birds were never acoustically isolated, but were visually isolated from one another during the experimental sessions by means of opaque barriers between cages. Following this study, birds were either kept for further experiments at Newcastle University or rehomed to outdoor aviaries.

### Pretrial Training

Birds were initially acclimatized to individual cages and foraging for food (domestic chick crumb) hidden in sand. This was done in order to remove any potential effects of differential neophobia to the apparatus or variation in the time course of a stress response to handling, and to achieve stable levels of food consumption prior to the experiment. Birds were already familiar with eating freely available crumb from ad libitum feeding in aviaries. We mixed food with sand to increase the amount of effort required to access the food. Starlings will forage for food in sand and previous experiments confirm that they are not able to assess food quantity in sand using the sense of smell (Bean et al., 1999). Previous studies of avian CFL have employed similar methods, requiring birds to search for food mixed with sand or wood shavings (Bean et al., 1999; Lindqvist & Jensen, 2008; Lindqvist et al., 2002, 2006; Schütz & Jensen, 2001).

Birds were caught from the aviary, weighed and placed individually in cages. Visual contact was maintained for the initial 3 days to aid habituation to cages, with barriers then placed between cages overnight (1700–0930 hours) to habituate birds to these prior to their use in experimental trials. Daily at noon following husbandry, we placed a bowl (18 cm diameter, 4 cm deep) containing 100 g of crumb (ad libitum quantity) and 200 g of sand into each cage, alternating on which side of the central divider the bowl was positioned. Birds also received four live mealworms (*Tenebrio* sp.) in a separate bowl. To obtain accurate food consumption measures, we sieved the crumb prior to use to remove dust or smaller pieces. We removed the bowl from the previous 24 h period and sieved the contents to collect the remaining crumb, which we weighed to measure food consumption. The training period continued until all eight birds ate at least 15 g of crumb in 24 h (maximum 9 days; mean 6.3 days).

### Experimental Phase

We measured CFL and free food consumption on 4 consecutive days because levels of CFL fluctuate within individual starlings (Bean et al., 1999). On the day preceding an experimental trial, we positioned barriers to visually isolate the birds at 1700 hours. On the morning of an experimental trial, we removed the ad libitum food bowl at 0800 hours, and weighed the remaining crumb to measure food consumption outside experimental trials (nontrial food consumption). Birds were then food deprived from 0800 to 1000 hours, after which we turned off the lights and placed two bowls in each cage. One bowl (CFL bowl) contained 39.5 g of crumb covered with 200 g of sand, with 0.5 g of crumb sprinkled on top of the sand. The other bowl (free food bowl) contained 200 g of crumb. The side position of the bowls was alternated between days. Trials began by switching on the lights once the experimenters had left the room. They lasted 2 h and were filmed using video cameras (Sony Handycam DCR-SR32) positioned outside the cages. At the end of the trial, any spilt food was placed in the appropriate bowl (determined by which side of the divider it was on) and both bowls were then removed. The remaining crumb was weighed to measure food consumption from each bowl (CFL consumption; free food consumption), following sieving of the CFL bowl to remove the sand. We then provided each cage with ad libitum food consisting of a bowl containing 200 g of crumb and four mealworms, and removed visual barriers until 1700 hours.

An observer blind to treatment scored by continuous observation of all trials on the video footage the duration of time spent in, perched on or with head over each bowl (CFL duration; free feeding duration). We used duration as a measure of foraging/CFL effort since this metric relates to the opportunity costs entailed. We measured these behaviours to confirm that foraging efficiency was lowered by the presence of sand in the CFL bowl, and because acquiring information about patch quality may involve not only ingesting food but also probing in sand without ingesting food (Bean et al., 1999; Lindqvist et al., 2002). Following the fourth trial, birds were caught and weighed. We used the mean of cage entry body mass and experiment end body mass for analyses of body mass. To examine whether hyperphagia occurred during trials, we summed for each day CFL consumption and free food consumption to give trial consumption. To examine whether hyperphagia occurred overall, we calculated for each day the mass of crumb consumed (total food consumption) in 22 h as the sum of trial consumption during the 2 h trial plus nontrial food consumption during the 20 h following the trial.

### Ethical Note

Our study adhered to the ASAB/ABS Guidelines for the Use of Animals in Research, and was approved by Newcastle University local ethical review committee. Work was conducted under U.K. Home Office project licence number PPL60/4073, and the removal of starlings from the wild was authorized by Natural England (licence number 20121066). Fieldwork was carried out with the permission of landowners, with the number and duration of nest disturbances minimized. One chick of 48 that we cross-fostered died between cross-fostering and the next morning; this is no greater than the expected rate of mortality this early in life. All other cross-fostered chicks gained weight between fostering and the next morning, suggesting rapid recovery from transport and acceptance in host nests. The manipulation was intended to increase developmental stress in the disadvantaged group. However, the level of within-brood size discrepancy created is within the natural range observed in starling nests (see Developmental Manipulation). Thus, the level of developmental stress was likely to have been within the naturally experienced range. The manipulation was also equally likely to improve a nestling's position within its nest as to make it worse. The mean weights for the disadvantaged birds were not significantly lower than for the advantaged birds at any weighing point during development; nor was the variance greater (Nettle, Monaghan, et al., 2015). Two disadvantaged birds and three advantaged birds died before day 12; this suggests that our disadvantageous manipulation did not result in excessive mortality and is in line with natural mortality rates in starlings (Feare, 1984). Sex was determined by molecular sexing as part of a related study using blood samples of 75 μl taken from the alar vein (see Nettle, Monaghan, et al., 2015). A small as possible needle was used and volume taken, and antiseptic cream was applied to the puncture site to minimize risk of infection. Stress due to catching adult birds was minimized by doing so in a darkened room using torchlight, with up to three persons catching simultaneously to minimize the time taken, and holding birds in cloth bags for the shortest possible time. The acute stress response is relatively short lived (Rich & Romero, 2005) and food consumption stabilized during the several days of pretrial training; thus variation in stress responses is likely to have had minimal impact on the experimental phase. The CFL experiment is likely to have induced short-term stress due to visual social isolation and the unfamiliar foraging environment. Birds were returned to their aviary within 22 days of removal. None showed any subsequent adverse effects.

### Statistical Analysis

From body mass measured after the final trial, we calculated body condition index as the residual mass corrected for tarsus length (mean of left and right tarsus measured on day 24, by which time the tarsus is fully grown) using an equation (body condition = mass −2.9158 × tarsus + 18.1278) derived from a simple regression of mass measured at day 115–123 (when birds were group-housed in aviaries for a sufficient period to obtain stable body mass) on day 24 tarsus. Residual condition indices offer a reasonable proxy for fat mass (Labocha & Hayes, 2012).

Data were analysed using general linear mixed models in R (R Development Core Team, 2011), using the base statistical procedures and package nlme (Pinheiro, Bates, DebRoy, Sarkar, & R Core Team, 2015). Model estimation was by maximum likelihood, and whether parameters differed significantly from 0 was determined by a likelihood ratio test (LRT) on the difference in model deviance (χ^2^ distributed) when the parameter was removed from the model. The degrees of freedom for this test equals the difference in the number of free parameters of the models that include and exclude the term of interest, which for the purposes of our study equated to 1 in all instances. We assumed a criterion for significance of *P* < 0.05 throughout; results with *P* < 0.10 are also reported and discussed as marginally nonsignificant trends.

We describe the main results relevant to the experimental hypotheses below and provide detailed specification of statistical models and output in Appendix Table A1, to which we refer by model number below. The raw data and R script are available as Supplementary Material. The response variables we examined were body mass (Appendix Table A1, Model 1), body condition index (Model 2), total food consumption (Model 3), trial consumption (Models 4 and 9), CFL consumption (Model 5), free food consumption (Model 6), CFL duration (Model 7), foraging rate (Model 8) and nontrial consumption (Model 10). The basic model for each response variable we studied included fixed effects for sex (since numbers were unbalanced between treatments), developmental treatment (ADV/DIS) and the sex*treatment interaction. Models included, where appropriate, nested random effects for individual bird identity (since the same individuals were measured for multiple days) and natal family (since quartets of birds were siblings). We did not include mass or body condition as model covariates in models of foraging behaviour since these could be consequences, rather than causes, of variation in foraging behaviour. The distribution of the outcome variables CFL consumption and foraging rate were zero inflated and truncated, requiring transformation to meet model assumptions. We experimented with different error structures and transformations and present here those giving satisfactory distribution of residuals (Gaussian error for all models; log transform of CFL consumption; square root transform of foraging rate). The sample size for all models was 37 birds.

## Results

### Mass and Body Condition

**Figure 1:**
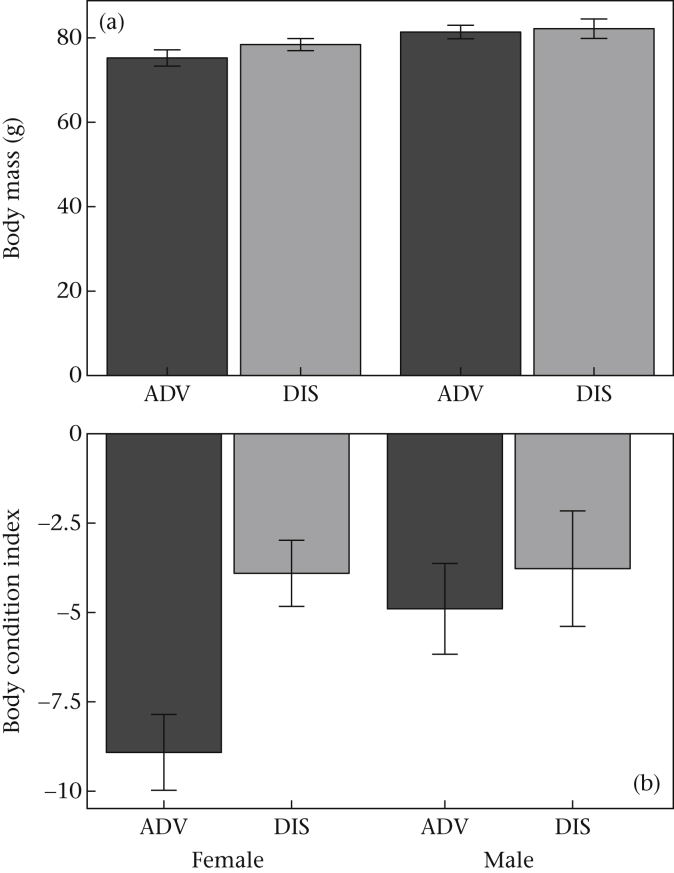
(a) Body mass, and (b) body condition index (residual mass) of developmentally advantaged (ADV) and disadvantaged (DIS) male and female starlings. Means ± 1 SE of raw data are shown.

We examined the effect of developmental treatment on body mass and condition (Models 1 and 2). ADV and DIS birds were of equivalent body mass at the time of the present experiment (Fig. 1a, Appendix Table A1, Model 1). Birds were, on average, lighter at the time of our experiment than when we calculated the body condition index equation (see Statistical Analysis); thus mean body condition indices were negative in our experiment (Fig. 1a). It is usual for birds to be lighter when individually caged than when in aviary groups (Feenders & Bateson, 2013), possibly due to reduced competition when alone (Witter & Swaddle, 1995). At the time of our experiment, DIS birds had significantly higher body condition indices than ADV birds (Fig. 1b, Appendix Table A1, Model 2).

### Food Consumption

**Figure 2:**
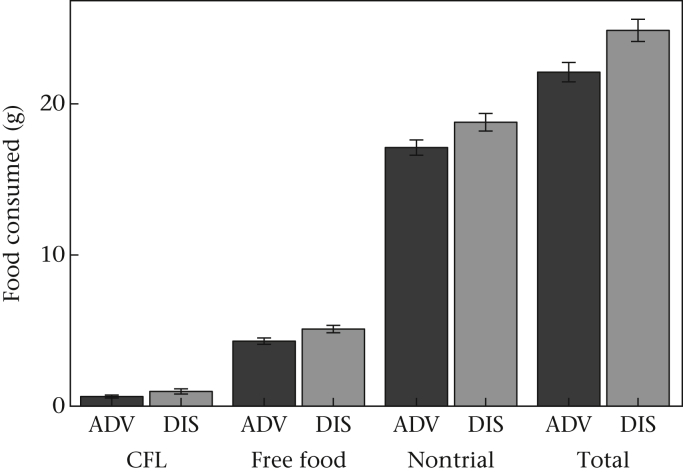
Mean mass of crumb consumed per day by developmentally advantaged (ADV) and disadvantaged (DIS) starlings from the contrafreeloading (CFL) bowl and free food bowl during the trial, the nontrial food consumption and total food consumption (i.e. the sum of the other measures). Means ± 1 SE of raw data are shown.

We compared the amount of food consumed by DIS and ADV birds both during trials (Model 4) and in total (Model 3) since treatment could potentially influence sensitivity to food deprivation which preceded the trials as well as overall hyperphagia. Total food consumption was marginally nonsignificantly greater for DIS birds than ADV birds (Fig. 2, Appendix Table A1, Cohen's *d* = 0.469, Model 3). There was a stronger effect of developmental treatment on trial consumption, with DIS birds eating significantly more food than ADV birds (Fig. 2, Appendix Table A1, Cohen's *d* = 0.691, Model 4).

Both DIS and ADV birds consumed more food from the free food bowl than by CFL (Fig. 2; greater free food consumption than CFL consumption in 132 of 148 trials). We compared CFL consumption (log transformed) of ADV and DIS birds while statistically controlling for free food consumption (Model 5). We included free feeding as a covariate because the amount consumed from the CFL bowl (assuming a fixed total energy requirement) is logically not independent from that at the alternative bowl. Indeed, CFL and free food consumption were negatively related for both ADV and DIS birds (estimate ± SE for free food consumption = −0.194 ± 0.029; Appendix Table A1, Model 5). DIS birds consumed marginally nonsignificantly more food by CFL than ADV birds (Fig. 2, Appendix Table A1, Model 5). Comparing free food consumption while controlling for CFL consumption as a covariate (Model 6), we found that DIS birds ate more free food than ADV birds (Appendix Table A1, Model 6).

### Foraging and Information Gathering Effort

**Figure 3:**
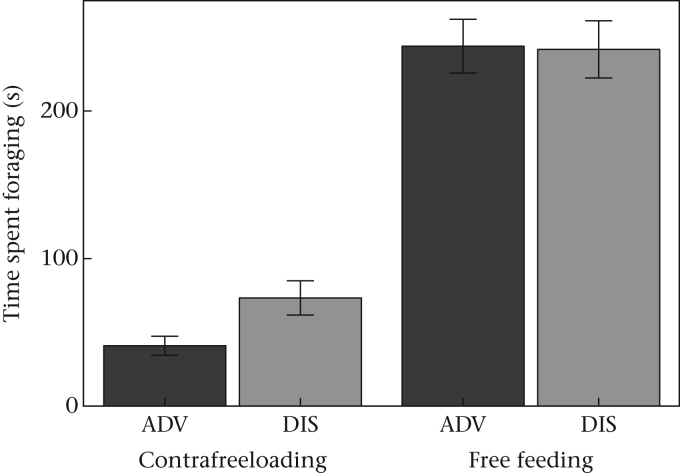
Foraging duration at free food and contrafreeloading bowls by advantaged (ADV) and disadvantaged (DIS) starlings. Means ± 1 SE of raw data are shown.

We assessed the effort put into foraging for food or information gathering in terms of the time spent at the free food bowl or CFL bowl, respectively. Both ADV and DIS birds spent more time foraging on freely available food than CFL (Fig. 3; mean free feeding duration > mean CFL duration at every trial for both ADV and DIS birds). We compared CFL effort of ADV and DIS birds in terms of the time spent foraging at the CFL bowl while statistically controlling for free feeding duration as covariate (Model 7). We did this because time spent on CFL (given the fixed trial length) is logically not independent from that at the alternative bowl. Indeed, CFL duration was negatively related to the time spent foraging on free food (estimate ± SE for free feeding duration = −0.127 ± 0.059; Appendix Table A1, Model 7). DIS birds spent marginally nonsignificantly more time on CFL than ADV birds (Fig. 3, Appendix Table A1, Model 7). There was a significant interaction between free feeding duration and treatment, indicating DIS birds spent more time on CFL for a given free foraging effort than ADV birds (Appendix Table A1, Model 7).

### Foraging Efficiency and Trade-offs

**Figure 4:**
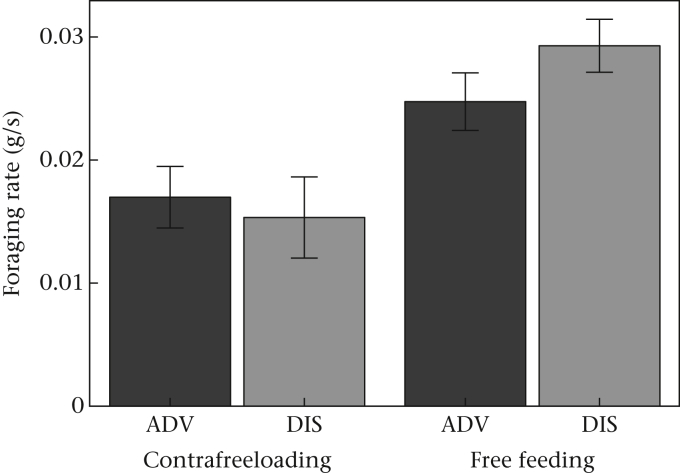
Foraging rate (food consumption per unit time spent at bowl) in contrafreeloading and free food bowls by developmentally advantaged (ADV) and disadvantaged (DIS) starlings. Means ± 1 SE of raw data are shown.

To confirm whether foraging was less efficient in the CFL bowl than when free feeding, as we had intended in our design, we used as a measure of foraging rate the food consumption per unit time (g/s) spent at each bowl (square-root transformed), including bowl (CFL or free food) as a fixed factor along with its interaction with treatment (Model 8). As intended, foraging in sand in the CFL bowl was less efficient than foraging in the free food bowl for both ADV and DIS birds (Fig. 4, Appendix Table A1, Model 8). On average, foraging in the CFL bowl resulted in a 40% reduction in foraging rate compared with free feeding. There was also a significant interaction between bowl and developmental treatment (Fig. 4, Appendix Table A1, Model 8). Although DIS birds foraged more efficiently than ADV birds on free food (mean DIS foraging efficiency > mean ADV foraging efficiency in three of four trials), the difference between treatments was not present for CFL (Fig. 4).

**Figure 5:**
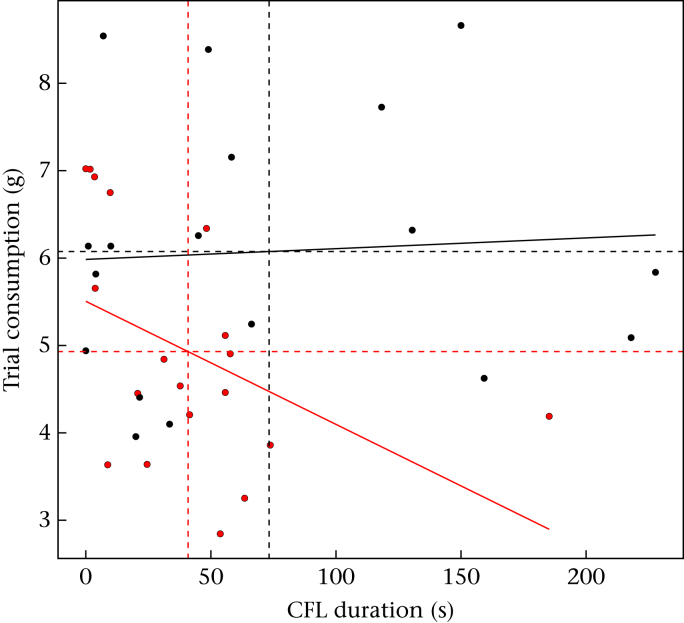
Trade-off between foraging and contrafreeloading (CFL) for developmentally advantaged (ADV) and disadvantaged (DIS) starlings. Linear regression lines are shown for ADV (red) and DIS (black) birds; data points are means per bird over the four trials. Dashed lines show mean trial food consumption or contrafreeloading duration for ADV (red) and DIS (black) birds.

We expected CFL to occur at the expense of a reduction in overall foraging success. To examine the trade-off between CFL and foraging success, we analysed trial consumption including CFL duration and its interaction with treatment as a covariate (Model 9). If a trade-off occurs then birds that spend more time on CFL would on average consume less food in total during the trial. The predicted trade-off between foraging and information gathering was present for ADV birds but not for DIS birds, as indicated by a negative relationship between trial consumption and the time spent on CFL for ADV birds only (Fig. 5, Appendix Table A1, Model 9). Since it is possible in our experiment that birds that spent more time on CFL during trials might compensate by consuming additional free food outside trials, we also analysed nontrial food consumption including CFL duration and its interaction with treatment as a covariate (Model 10). There was no relationship between nontrial food consumption and time spent on CFL (Appendix Table A1, Model 10).

## Discussion

Early life competitive disadvantage exerted a long-lasting influence on starlings' motivation to forage for food and gather information about food sources. Despite the absence of an effect of developmental treatment on early growth (Nettle, Monaghan, et al., 2015), developmentally disadvantaged birds were fatter in adulthood at the time of our study than advantaged birds (Fig. 1) and showed differences in their foraging behaviour. Disadvantaged starlings were hyperphagic following food deprivation (Fig. 2) and foraged at a faster rate on freely available food (Fig. 4) than their advantaged siblings. They also spent more time gathering information about food via CFL (Fig. 3), and consumed marginally more food by CFL, than advantaged birds (Fig. 2). The expected trade-off between foraging and CFL was confirmed for advantaged starlings, since those that contrafreeloaded more also consumed less food during trials (Fig. 5). However, there was no trade-off for disadvantaged birds, probably because of their higher foraging efficiency on freely available food. We predicted that developmentally disadvantaged starlings would contrafreeload more than advantaged birds, this being a means to gather information that could serve as insurance against future changes in food availability. As expected, we found that disadvantaged birds spent more time on CFL and consumed marginally more food by CFL than advantaged birds. Also as predicted, disadvantaged birds consumed more food than advantaged birds, but significantly so only following food deprivation. Disadvantaged birds also carried more energy reserves (fat), as expected. Thus developmentally disadvantaged starlings appeared to employ both energy storage and information gathering (CFL) foraging strategies to a greater extent than advantaged birds. Since our manipulation had no detectable impact on the timing of weight gain, or weight or wing length at fledging (Nettle, Monaghan, et al., 2015), the impact of developmental history on foraging and information gathering in our birds as adults appears not to be wholly due to variation in the overall pattern of growth (i.e. to food insufficiency). Our study thus demonstrates that early life disadvantage can have long-lasting effects on foraging and mass regulation even in the absence of food insufficiency during development. Instead, psychosocial stress and/or food insecurity resulting from competition in early life appears to have been sufficient to cause these differences. Below, we defend our measure of CFL then discuss our results in terms of adaptive developmental plasticity, and consider the extent to which our findings support the idea that subtly developmentally disadvantaged birds show a ‘memory of hunger’ (Bloxham et al., 2014).

Several lines of evidence support the assumption that our experiment measured CFL, that is, working for food when equivalent food is freely available, in order to gather information. First, foraging in sand entailed a 40% reduction in food intake rate compared with free feeding, as expected if work was entailed. Second, at least for advantaged birds, the more time a bird spent searching for food in sand, the less food it consumed during a trial; thus CFL carried a foraging opportunity cost. As with laboratory studies generally, it is difficult to translate such a deficit into a fitness cost. In our experiment, birds that spent more time on CFL did not appear to compensate for lost foraging opportunity by increasing food intake outside trials when only free food was available. In the wild, CFL opportunities may be present throughout the day, so the trade-off against free feeding could have potentially greater impact on overall energy intake. Third, a previous study found that starlings search for food in sand more if the surface is visually occluded, suggesting that information gathering is a driver (Bean et al., 1999) rather than the behaviour simply representing exploration or activity per se without information gain. CFL may in fact be a form of exploration since exploration has been considered as a means to gain information about the environment (Inglis, Langton, Forkman, & Lazarus, 2001).

Unlike rodent models of early life adversity, our disadvantaged starlings were not strongly hyperphagic overall, since their daily food consumption was only marginally increased compared with their advantaged siblings. Disadvantaged birds did show significant hyperphagia, however, following 2 h of food deprivation. Hyperphagia in rats is especially pronounced for very high-quality foods (Vickers et al., 2000), whereas in the current study starlings were foraging on a lower quality (low-protein) food. Consistent with this explanation for limited hyperphagia overall, in a previous study, starlings raised in enlarged brood sizes were hyperphagic only for high-protein foods (mealworms) and not for a lower-protein (crumb) diet (Bloxham et al., 2014). To elucidate the factors influencing hyperphagia, future work should examine interactions between current energetic state and reserves, dietary quality and developmental history on foraging and information gathering.

As evidenced by their greater foraging efficiency on free food as well as increased CFL effort, developmentally disadvantaged starlings showed greater motivation for both foraging for food and information gathering. Thus they appear to retain a phenotypic ‘memory’ of increased early food competition. Increased foraging in response to current food restriction has been reported in a range of species, including humans (Garner, 1997; Redman & Ravussin, 2011; Vitousek, Manke, Gray, & Vitousek, 2004). Increased body mass index is also associated with current food insecurity in humans (Adams, Grummer-Strawn, & Chavez, 2003; Martin-Fernandez, Caillavet, Lhuissier, & Chauvin, 2014; Townsend, Peerson, Love, Achterberg, & Murphy, 2001). Our study adds to this by demonstrating a lasting effect of prior disadvantage in food competition on adult foraging behaviour and energy reserves. The results are consistent with our previous study in which we found that starlings reared in enlarged broods also showed a phenotypic memory of hunger, in the form of reduced dietary selectivity of toxic prey (Bloxham et al., 2014). From an adaptive perspective, it may be beneficial for animals faced with high competition and/or low food reliability to increase their foraging activity and information gathering, thus increasing the relative priority of getting food and information concerning sources of food over other competing activities. Wild animals often reduce the performance of maintenance behaviours, play and affiliative behaviour when food is scarce (D'Eath, Tolkamp, Kyriazakis, & Lawrence, 2009). It remains to be investigated what cost, if any, such foraging prioritization may entail for disadvantaged starlings. Possibilities warranting further study include reduced vigilance or feather maintenance.

In terms of CFL, disadvantaged starlings behaved unlike birds experiencing acute current food restriction, the latter reducing CFL following 8 h of food deprivation (Bean et al., 1999). Reduction in CFL has been interpreted as an energy-conserving strategy (Lindqvist et al., 2002). Therefore the difference may be because our developmental treatment is likely to have altered perceptions of food uncertainty as opposed to birds' current energy intake requirement, as birds in our study were minimally food deprived (2 h) during trials and disadvantaged birds were equal in body mass and fatter than their advantaged siblings. The greater CFL by disadvantaged birds might alternatively be in part a consequence of their apparent higher foraging motivation overall. However, motivational differences cannot completely account for the greater CFL by disadvantaged birds since even for a given free foraging effort, disadvantaged birds spent more time on CFL. Since disadvantaged birds spent both absolutely and proportionally more time on CFL than their advantaged siblings they invested more in information gathering directly. An alternative nonadaptive possibility is that disadvantaged birds spent more time on CFL (and as a result consumed marginally more contrafreeloaded food), owing to a reduced learning capacity making them less efficient in acquiring information about the foraging patch. Learning performance is reduced in zebra finches, *Taeniopygia guttata*, experiencing low-quality early nutrition (Brust, Krüger, Naguib, & Krause, 2014). Reduced learning ability has been suggested to explain the reduced CFL of rats reared in enriched environments (Coburn & Tarte, 1976). However, we consider an explanation based on differential learning ability unlikely since independent measures of learning did not differ between these same advantaged and disadvantaged birds (Nettle, Andrews, et al., 2015).

Under an adaptive developmental plasticity framework, it is possible that our disadvantaged birds were less robust to food deprivation in a way not captured by our gross measures of body mass or condition. That hyperphagia was more pronounced in disadvantaged birds following food deprivation is at least consistent with this. Within-brood competition (Verhulst, Holveck, & Riebel, 2006) and early exposure to the stress hormone corticosterone (Spencer & Verhulst, 2008) can raise adult metabolic rate. Thus, is it possible that disadvantaged birds have a differing metabolic requirement as adults, perhaps altering their sensitivity to food shortages and hence making information about alternative foraging patches more valuable to them or less costly to obtain, or reducing the utility of a given level of reserves (Mathot & Dall, 2013). Other cryptic physiological differences are possible, for example, physiological constraints such as digestive capacity can be influenced by feeding history in nestlings (Wright et al., 2002), although the persistence of such effects into adulthood is unknown.

The optimal level of sampling the environment to gain information and hence reduce uncertainty over food supply theoretically depends on the relative costs of missing good foraging opportunities versus the costs of sampling, and the rate at which the environment changes. These factors may be influenced by developmental history, suggesting an alternative adaptive explanation for increased CFL by disadvantaged starlings. Birds experiencing a poor start may be disadvantaged in social competition as adults owing to a lower phenotypic quality, making their foraging environment effectively more uncertain. Experimentally elevated corticosterone in zebra finch nestlings influences social network position in adulthood (Boogert, Farine, & Spencer, 2014). Disadvantaged birds could possibly be more vulnerable to food unpredictability induced through competition, again making information on alternative food sources more valuable to them. In the wild, developmental disadvantage could be predictive of a more uncertain environment per se independent of competition, perhaps selecting for developmentally plastic strategies to insure against or reduce uncertainty via sampling the environment by CFL. In opposition to these hypotheses, CFL has been shown to be reduced by increased environmental uncertainty (Forkman, 1991) as well as by increased effort to obtain earned food (Rutter & Nevin, 1990), both of which are likely to result from inferiority in social competition.

Our sibling birds experienced only 10 days of differential experience as nestlings while living their subsequent lives in the same environment, and did not differ in early growth. Even such limited difference in exposure to competition appears sufficient to have multiple, long-lasting effects: not only did it impact a biomarker of stress (telomere attrition; Nettle, Monaghan, et al., 2015) and flight performance (O'Hagan et al., 2015), it also induced increased motivation to forage for food and gather information about sources of food and, perhaps consequently, to carry more body fat. Thus, our findings add to previous evidence in birds and other taxa (Bateson, Brilot, Gillespie, Monaghan, & Nettle, 2015; Bloxham et al., 2014; Boogert et al., 2014; Boogert, Zimmer, & Spencer, 2013; Criscuolo, Monaghan, Nasir, & Metcalfe, 2008; Felitti et al., 1998; Lindström, 1999; Nettle, Monaghan, et al., 2015; Nowicki, Searcy, & Peters, 2002; O'Hagan et al., 2015; Tschirren, Rutstein, Postma, Mariette, & Griffith, 2009; Verhulst et al., 2006; Zimmer, Boogert, & Spencer, 2013) that even subtle early life manipulations can induce enduring alterations in physical and behavioural phenotypes. The question why such lasting influences of early environment endure into adulthood even when current energetic or environmental conditions apparently no longer differ has been the topic of much theoretical debate (Dall et al., 2012; Frankenhuis & Panchanathan, 2011). The effects in adulthood may be a nonadaptive consequence of behaviour that was advantageous during the nestling or immediate postfledging period when selection is most intense. Instead, we may plausibly interpret the altered phenotype as remaining beneficial in adulthood for birds that experienced a poor start in life, perhaps due to reduced certainty over the future availability of, or access to, foraging patches. To this end, the social dominance of developmentally disadvantaged birds and their relative foraging success in unpredictable food environments remains to be examined. Understanding the underlying causes of individual variation in foraging behaviour and resource or information acquisition may help us to elucidate behavioural variation typically thought of as personality variation (Dall et al., 2012) and could improve our understanding of the ontogeny of food-related conditions such as obesity in humans (Cottrell & Ozanne, 2008).

## Figures and Tables

**Table A1:** Output of statistical models

Model no.	Response variable	Random effects	Fixed effects	LRT	*P*
Mass and body condition					
1	Body mass	Natal nest	Sex^f^	11.62	0.001
Treatment^f^	1.77	0.183
Sex*Treatment	0.16	0.685
2	Body condition index	Natal nest	Sex^f^	5.32	0.021
Treatment^f^	6.83	0.009
Sex*Treatment	0.02	0.893
Food consumption					
3	Total food consumption	Natal nest/Bird	Sex^f^	8.73	0.003
Treatment^f^	2.98	0.085
Sex*Treatment	0.00	0.9500
4	Trial consumption	Natal nest/Bird	Sex^f^	7.18	0.007
Treatment^f^	6.27	0.012
Sex*Treatment	0.01	0.927
5	log(CFL consumption)	Natal nest/Bird	Sex^f^	7.11	0.008
Treatment^f^	3.79	0.052
Sex*Treatment	0.39	0.531
Free food consumption^c^	61.07	<0.001
Free food consumption*Treatment	2.12	0.147
6	Free food consumption	Natal nest/Bird	Sex^f^	6.66	0.010
Treatment^f^	5.79	0.016
Sex*Treatment	0.00	0.948
CFL consumption^c^	62.18	<0.001
CFL consumption*Treatment	3.10	0.078
CFL effort					
7	CFL duration	Natal nest/Bird	Sex^f^	0.31	0.577
Treatment^f^	3.45	0.063
Sex*Treatment	0.43	0.510
Free feeding duration^c^	26.97	<0.001
Free feeding duration*Treatment	6.16	0.013
Foraging efficiency					
8	√Foraging rate	Natal nest/Bird	Sex^f^	4.60	0.032
Treatment^f^	0.04	0.838
Sex*Treatment	0.00	0.998
Bowl^f^	46.33	<0.001
Bowl*Treatment	5.16	0.023
Trade-off between CFL and foraging success					
9	Trial consumption	Natal nest/Bird	Sex	7.06	0.008
Treatment	5.47	0.019
Sex*Treatment	0.00	0.962
CFL duration^c^	1.42	0.234
CFL duration*Treatment	6.00	0.014
10	Nontrial food consumption	Natal nest/Bird	Sex	8.64	0.003
Treatment	1.29	0.256
Sex*Treatment	0.01	0.915
CFL duration^c^	1.53	0.216
CFL duration*Treatment	0.94	0.332

This table provides the output from the statistical models described in the Results. All models are general linear mixed with Gaussian error structure, run using package ‘nlme’ in R. LRT is the likelihood ratio test statistic comparing models with and without the fixed effect of interest (see Statistical Analysis); *P* is the *P* value for this likelihood ratio test. All fixed and random effects included in the model are shown. All models included a random nest-of-origin effect (Natal nest); where the data contained multiple measurements from individual birds a random effect of individual (Bird) nested in Natal nest was also included. Body mass is the mean of cage entry body mass and experiment end body mass. Body condition is the residual mass corrected for tarsus length (see Statistical Analysis for details). Total food consumption is the daily summed mass of food (g) consumed outside the experimental trial (nontrial consumption) and during the trial (trial consumption) from both free food and CFL bowls. CFL consumption and free food consumption is the mass of food (g) consumed from the CFL bowl or free food bowl, respectively during a daily trial. Trial consumption is the sum of CFL consumption and free food consumption. CFL duration and free feeding duration are the daily times (s) spent in, perched on or with head over the CFL or free food bowl, respectively. Foraging rate is the food consumption per unit duration (g/s) i.e. CFL consumption/CFL duration or free food consumption/free feeding duration. The fixed factor treatment refers to whether a bird experienced the ADV or DIS developmental manipulation of early life competition as a nestling. The fixed factor Bowl (Model 7) refers to foraging carried out in the free food or CFL bowl. ^f^Denotes a fixed factor; ^c^denotes a covariate.
